# Investigating regionalization techniques for large-scale hydrological modelling

**DOI:** 10.1016/j.jhydrol.2018.12.071

**Published:** 2019-03

**Authors:** Liliana Pagliero, Fayçal Bouraoui, Jan Diels, Patrick Willems, Neil McIntyre

**Affiliations:** a Institute for Environment and Sustainability, Joint Research Centre, European Commission, Ispra I-21027, Italy; b Dept. Civil Engineering, Hydraulics Section, KU Leuven, Leuven B-3001, Belgium; c Dept. Earth and Environmental Sciences, Division of Soil and Water Management, KU Leuven, Leuven B-3001, Belgium; d Sustainable Minerals Institute, The University of Queensland, Brisbane, Australia

**Keywords:** Clustering, Hydrological modelling, Large-scale, Regionalization, Similarity, SWAT

## Abstract

This work investigates regionalization techniques for large-scale model applications in the frame of a pan-European assessment of water resources covering approx. 740,000 km^2^ in Western Europe. Using the SWAT platform, four variants of the similarity based regionalization approach were compared. The first two involved unsupervised clustering to define hydrological regions before performing hydrological model calibration, whereas the last two involved supervised clustering after performing calibration. Similarity is defined using Partial Least Squares Regression (PLSR) analysis that identifies watershed physiographic characteristics that are most relevant for the selected hydrological response indices.

The PLSR results indicate that typically available watershed characteristics such as geomorphology, land-use, climate, and soil properties describe reasonably well the average hydrological conditions but poorly the extreme events. Regionalization variants considering unsupervised clustering and supervised clustering performed similarly well when using all available information. However, results indicate that supervised clustering uses data more efficiently and may be more suitable when data are scarce. It is demonstrated that parsimonious use of available data can be achieved using both regionalization techniques. Finally, model performance consistently becomes acceptable by calibrating watersheds covering only 10% of the model domain, thus, making the calibration task affordable in terms of time and computational resources required.

## Highlights

• Alternative regionalization techniques in large-scale model applications were investigated.
• Regionalization techniques are model-independent and are based on the similarity approach.
• Adapted measures of physiographic similarity meaningful for hydrological similarity were used.
• Regionalization involving unsupervised and supervised clustering were studied.
• These techniques proved to be helpful in large-scale model calibration.

## 1 Introduction

Hydrologic and water quality models have emerged as essential tools for successful implementation of regional to continental scale river basin planning initiatives, such as the European Water Framework Directive (WFD) (EC., 2000, Hartnett et al., 2007). The ambitious spatial scale of these models and multiple modellers and stakeholders involved exacerbate the challenges inherent to environmental modelling, including: reaching agreement on modelling objectives; selection of suitable models; assessment of data quality; model parameter estimation; model assessment; reporting of model uncertainty and assumptions; and review and auditing processes. This calls for frameworks that support uniform implementation of model procedures and clarity on the modelling decisions taken. This paper addresses this by developing model parameter estimation and assessment procedures suitable for pan-European hydrological modelling, focusing on ungauged watersheds where conventional model calibration approaches are not applicable.

While much of Europe benefits from a large number of gauged watersheds, combining data from the Global Runoff Data Centre (GRDC), the European Water Archive (EWA) and national databases, the principal parameter estimation problem in a pan-European modelling exercise is that of ungauged watersheds. Although some models attempt to include physics-based parameters, such as channel slopes, which may be directly estimated from measured properties of a watershed, it is generally agreed that acceptable model accuracy can only be achieved by empirical estimation of at least some of the parameters, which for ungauged catchments is approached via regionalization (Prediction in Ungauged Basins (PUB) initiative) (Wagener and Montanari, 2011, Sivapalan, 2003, Winsemius et al., 2009, Blöschl et al., 2013, Hrachowitz et al., 2013). A large number of hydrological model regionalization methods have been proposed in the literature. All of these are based around the concept that information about hydrological response can be transferred between watersheds that may be assumed to be hydrologically similar, usually based on knowledge of their relevant properties such as land cover. The differences between approaches lie in the type of information that is transferred, the transfer method and the watershed properties used to quantify similarity.

The most common approach is regression of model parameters that are calibrated at gauged (donor) watersheds against the corresponding watershed characteristics. The regression model can then be used to estimate the hydrological model parameters at any ungauged watershed for which the same characteristics are known. Typically, linear regressions are used for each model parameter independently (Heuvelmans et al., 2006, Gitau and Chaubey, 2010). Other more sophisticated relationships, including non-linear, weighted and sequential regressions, have also been applied (Kay et al., 2006, Abdulla and Lettenmaier, 1997, Li et al., 2010). Where a large number of inter-related characteristics are potentially relevant, their principal components have been used as inputs to regression (Livneh and Lettenmaier, 2013). With the increasing use of continental-scale models remote sensed data (leaf area index, canopy height, among others) has increasingly been relied upon (Chaney et al., 2016). Although widely used, the regression approach has major limitations, including that model parameters may not be strongly related to measurable attributes, and further, parameter inter-dependency is often too complex to encompass satisfactorily in the regression or overlooked.

A second group of approaches avoids regression by transferring the entire set of information about hydrological response between watersheds. Commonly this is implemented in the following way. For each ungauged watershed, a donor watershed is identified that is most similar in terms of watershed characteristics, and the ungauged watershed then receives the complete parameter set calibrated for the donor watershed. If it is considered that there is some uncertainty about the ‘most similar’ donor watershed, the approach may be extended to identifying clusters of similar watersheds (Bormann et al., 1999, Bormann, 2010, Ramachandra Rao and Srinivas, 2006) and transferring ensembles of hydrological models within the cluster (McIntyre et al., 2005, Reichl et al., 2009, Drogue and Khediri, 2016). The primary advantage of this approach is that the model parameter inter-dependencies are maintained rather than being simplified to facilitate regression (Parajka et al., 2005). A simplification of this ‘transfer’ approach is where spatial proximity between watersheds is used as a proxy of similarity, sometimes implemented within a kriging framework (Skøien et al., 2006, Patil and Stieglitz, 2012. A drawback of these methods is the question of whether or not similar watersheds tend to occur in close proximity to each other (Parajka et al., 2005, Wagener and Wheater, 2006). An alternative to either regression or similarity-based approaches is spatial interpolation of calibrated parameters. Although spatial complexity is likely to affect applicability, it has been found to be an efficient approach for large-scale applications (Troy et al., 2008).

As can been seen from the examples cited in this review, both the regression and transfer approaches have been applied to a range of hydrological model types, ranging from simple conceptual type models to highly parameterised land surface models.

Either the regression or transfer approaches may be combined with regional-scale calibration. Two main methods have been used. First, the calibration of the parameters of the hydrological model can be combined with estimating the parameters of the regression (Fernandez et al., 2000, Parajka et al., 2007), which sacrifices performance at gauged locations in order to optimise the precision of the regression model. Second, all gauged watersheds that fall within a region (e.g. Viney et al., 2015) or cluster of similar watersheds (e.g. Szolgay et al., 2003) may be calibrated simultaneously to produce a parameter set that is applicable across all watersheds in the region or cluster.

Inter-comparisons of approaches have not identified one that consistently performs better (Parajka et al., 2005, McIntyre et al., 2005, Kay et al., 2006, Ayzel et al., 2017). Oudin et al. (2008) suggested that this may be due to differences in data types, differences in the selected watershed characteristics, and differences in model structures. For pan-European applications such as this in the context of the EU initiative “Blue Print to Safeguard Europe’s Water Resources” (EC, 2012), the clustering of watersheds according to expected hydrological similarity and the transfer of model parameter sets within clusters may be preferred – The large range of watershed types involved and the large number of parameters mean that the regression-based methods are not considered promising. Instead, priority research questions relate to watershed clustering strategies that support the transfer of parameter sets to ungauged watersheds, and identifying information gaps that limit the success of the strategy.

When using the similarity approach, the problem lies in how to identify hydrologically similar watersheds, i.e., watersheds with similar response to precipitation input, quantified through hydrological signatures that are relevant to the prediction context (Sawicz et al., 2011). Typically regionalization studies assume that watersheds that have apparently similar physical characteristics have similar hydrological behaviour. For example, Reichl et al. (2009) identified as similarity measures commonly used watershed attributes based on geomorphology, vegetation cover, climate and soil properties, although with considerable uncertainty about which were the optimal characteristics. Oudin et al. (2010) compared pools of hydrologically similar watersheds with pools of apparently physically similar watersheds concluding that a significant overlap occurred only for 60% of the watersheds. Furthermore, recent studies suggest that similarity in watershed streamflow response is dynamic and highly dependent on flow conditions (Patil and Stieglitz, 2011). Therefore, the selection of physical characteristics upon which to base the similarity measure is a challenging part of the ungauged catchment problem.

Following from this, in the context of pan-European hydrological modelling, the aims of this paper are: 1) Develop an objective procedure for selecting watershed properties that are relevant for defining clusters of hydrologically similar watersheds; 2) Compare approaches to clustering watersheds and transferring hydrological model parameter sets within these clusters, in terms of model performance from small watershed up to continental scales; 3) Identify the primary information gaps that increase variance within clusters and limit ungauged watershed performance.

## 2 Materials and methods

### 2.1 Description of the study area

**Fig. 1:**
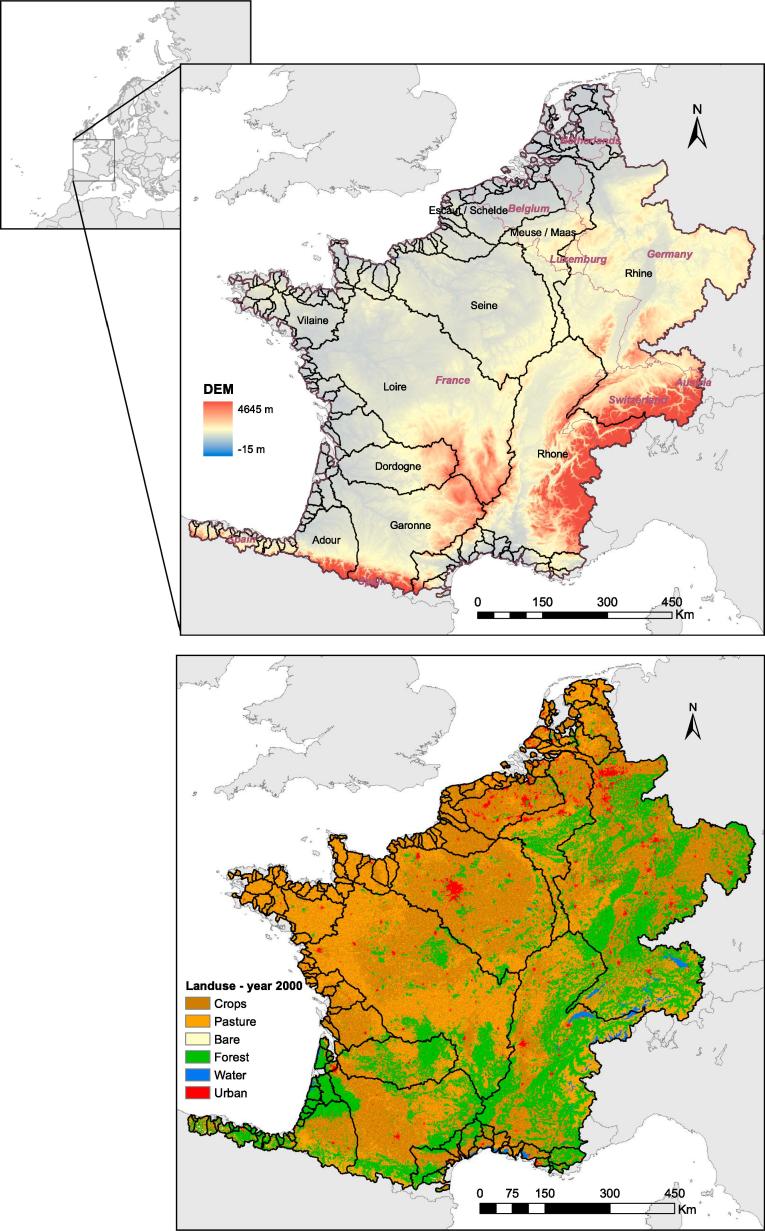
Overview modelled area.

The region modelled is located in Western Europe and covers an area of 736,780 km^2^ (see Fig. 1). The most important river basins, in terms of drainage area, include the Rhine (160,221 km^2^), the Loire (116,981 km^2^), the Rhone (96,619 km^2^), the Seine (75,990 km^2^), the Garonne (55,703 km^2^), the Meuse (32,047 km^2^) the Dordogne (23,902 km^2^), the Scheldt (18,949 km^2^), the Adour (16,861 km^2^) and the Vilaine (10,490 km^2^). The modelled area covers France, Belgium and Luxemburg, an important part of Switzerland, half the Netherlands, one third of Germany and parts of Austria, Spain, and Italy.

### 2.2 Hydrological modelling platform

In the context of the Blue Print, there is a need for a common, uniformly applied hydrological model, to support a common understanding of errors, assumptions and limits of applicability. There is also a need for a model that allows links between land and water management and output variables, in terms of a range of hydrological and water quality variables, to be simulated. SWAT (Arnold et al., 1998, Arnold and Fohrer, 2005) was selected as a modelling platform that can potentially meet these requirements.

SWAT is a basin scale, semi-distributed, conceptual model that operates on a continuous time scale with a daily time step. SWAT subdivides a basin into subwatersheds, which are connected by a stream network. Subwatersheds can be further divided into Hydrologic Response Units (HRUs), which are unique combinations of land use, soil type and slope. Hydrologic simulation is based on the water balance equation and is separated into two major components: the land phase, which simulates the amount of water, sediment, nutrient, and pesticide loadings to the main channel of each subwatershed, and the routing phase, which simulates the movement of water, sediments, and nutrients through the watershed channel network to the outlet (Neitsch et al., 2009).

Within the land phase, SWAT simulates the processes of snow fall and melting and the build-up of the subflows that compose the streamflow, namely, baseflow, interflow, and overland flow. The plant growth simulation submodel, within the SWAT land cover component, is based on the EPIC plant growth model (Williams, 1995).

### 2.3 Model input

The model was set up using readily available datasets. For the geographical data, Chaplot, 2005, Bormann et al., 2009 have shown that the SWAT model results may be significantly affected by the spatial resolution of these data. The resolutions considered next are based on a trade-off between the resolutions at which the data are readily available at the pan-European level, and the size of the subwatersheds considered (as discussed further in this section).

• A Digital Elevation Map (DEM) at 100 × 100 m resolution was obtained from the Shuttle Radar Topography Mission (SRTM).
• A land use map at 1 × 1 km for the year 2000 was built from the combination of the Common Agricultural Policy Regionalized Impact modelling system (CAPRI) (Britz, 2004), the center for Sustainability And Global Environment (SAGE) (Monfreda et al., 2008), the History Database of the global Environment (HYDE 3) (Klein Goldewijk and van Drecht, 2006) and the Global Land Cover (GLC2000) (Bartholomé and Belward, 2005) databases.
• A soil map at 1 × 1 km was obtained from the Harmonized World Soil Database (HWSD) (FAO, IIASA, ISRIC, ISS-CAS, JRC, 2008). The soil parameters required in this study were adapted directly from the HWSD and calculated using pedotransfer functions for saturated hydraulic conductivity (Wösten et al., 1998) and for the Universal Soil Loss Equation (USLE) soil erodibility factor (Williams, 1995).
• Subwatershed and stream delineation corresponds to the HydroEurope Geodatabase developed by Bouraoui and Aloe (2009) based on the Catchment Characterization Modelling version 2 (CCM2) database for continental Europe (Vogt et al., 2007).
• Data regarding 33 reservoirs and lakes with an area larger than 20 km^2^ were obtained from the Global Lakes and Wetland Database (GLWD) (Lehner and Döll, 2004) and the CCM2 database (Vogt et al., 2007).

Climate data used in this study included daily precipitation, temperature, solar radiation, wind speed and relative humidity obtained from the Monitoring Agricultural ResourceS MARS (Rijks et al., 1998) meteorological database, which is a gridded data set 25 × 25 km.

Discharge data used for parameter calibration and validation were collected from a number of sources, including the Global Runoff Data Centre (GRDC), the Flemish Environmental Agency, EauFrance and the General Directorate of Waters of the Ministry of Environment of Spain, that after quality and consistency examination resulted in 698 stations with daily data for the period 1980–2009 or sub-periods therein.

### 2.4 Model setup

The model was implemented in SWAT2009 using the ArcGIS SWAT interface (Winchell et al., 2010). It includes the river basins mentioned earlier, as well as neighbouring coastal basins. The total modelled area is 736,780 km^2^ and it was divided, based on the CCM2 database, into 4,216 subwatersheds with an average size of 175 km^2^. The subwatersheds were defined using dominant soil, land use types and slope; i.e. only one HRU was determined within a subwatershed. These subwatersheds were considered to be an appropriate modelling unit because this resolution is an adequate trade-off between model detail and computational resources needed to model at continental scale. Elevation bands were used in steep subwatersheds to represent snow and precipitation gradients. From hereafter, we refer to “subwatersheds” as the smallest modelling unit.

Irrigated areas were defined by overlaying the land use map and the FAO global irrigation map (Siebert et al., 2007). Management practices, that include auto-irrigation and fertilization, were defined as fractions of potential heat unit requirements of the crops. Heat units were calculated for each crop using local climate data. Fertilizer applications were obtained for the year 2000 from CAPRI (Britz, 2004). Management practices were adjusted and validated by controlling the optimal growth of the crops and by comparing simulated crop yields with country statistics extracted from Eurostat agricultural database (official national statistics reported by Member States to the European Commission).

The model includes only reservoirs and lakes with areas larger than 20 km^2^, and other hydraulic influences have not been included.

### 2.5 Calibration

A step-wise calibration protocol was applied, as described in Pagliero et al. (2014). Step-wise calibration was implemented by separating the watershed response output into the three main flow components: baseflow, interflow and overland flow. Snow melt was also calibrated by adjusting the timing of the hydrograph. Parameters were calibrated sequentially through the flow components.

Initial parameter values derived through the ArcSWAT interface were used as starting points for the optimization. ArcSWAT extracts, for every HRU and/or subwatershed, topographic parameters from the DEM map, assigns parameter values of soil type and land use by clipping soil type and land use input maps, and matches subwatersheds and weather stations based on location (Di Luzio et al., 2004, Olivera et al., 2006). Model parameters, included those calibrated are presented in Section A.1 in the appendices.

Calibration was performed in 246 selected head subwatersheds. The selection criteria were: they are head subwatersheds, i.e. there are no upstream subwatersheds and flow monitoring data is available for 1995–2004. This period was selected because it corresponds to the most complete consecutive period of flow data available (see Section 2.3). Using head subwatersheds, as well as avoiding the need to calibrate two or more subwatersheds together, limited the influence that hydraulic structures and other regulation strategies and man-made influences have on the river flows.

A combination of manual and automated (SUFI-2; Abbaspour et al., 2004) calibration was applied. The efficiency criterion was bR^2^, which is a combination of R^2^ and the gradient b of the regression between measured and predicted daily flow on which R^2^ is based. By weighting R^2^ by b, under- or over-predictions are quantified together with the correlations (Krause et al., 2005).

For a more comprehensive characterization of the performance, the Normalized Root Mean Square Error (NRMSE%) and the Percent Bias (PBIAS%), which measures the average tendency of the simulated values to be larger (positive PBIAS) or smaller (negative PBIAS) than the observed ones, were also calculated. Given the aim of the model to support water quality management, for which both short and longer duration flows are important, monthly flow performance was assessed as well.

### 2.6 Defining watershed similarity

In this work, regionalization is based on the similarity approach. Physiographic watershed characteristics were selected that can be derived for the entire study area and that are potentially relevant for explaining the hydrological response. To capture a broad spectrum of flow conditions, a variety of discharge characteristics were considered (see Table 1).

**Table 1:** Dependent (discharge characteristics) and independent (watershed characteristics) variables for PLSR.

Watershed characteristics (X)	Discharge characteristics (Y)
1. River length [km]	1. Mean annual discharge [mm yr^−1^]
2. Maximum elevation [m]	2. Coefficient of variation of annual discharge [−]
3. Minimum elevation [m]	3. Coefficient of variation of daily discharge [−]
4. Average elevation [m]	4. Coefficient of variation of annual minima [−]
5. Median slope [%]	5. Coefficient of variation of annual maxima [−]
6. Clay content [%]	6. Daily discharge with pbbexc* 90 [mm]
7. Sand content [%]	7. Daily discharge with pbbexc* 70 [mm]
8. Shrub area [%]	8. Daily discharge with pbbexc* 50 [mm]
9. Bare soil area [%]	9. Mean annual baseflow [mm yr^−1^]
10. Forest area [%]	10. Mean annual quickflow [mm yr^−1^]
11- Water area [%]	11. Baseflow/Discharge summer [−]
12. Urban area [%]	12. Baseflow/Discharge winter [−]
13. Cropland area [%]	13. Baseflow/Rainfall [−]
14. Grassland area [%]	14. Quickflow/Rainfall [−]
15. Annual precipitation [mm yr-1]	
16. Average maximum temperature [°C]	
17. Average minimum temperature [°C]	
18. Annual potential evapotranspiration [mm yr-1]	
19. Average number of days with precipitation	

* pbbexc: probability of exceedance.

Partial Least Squares Regression (PLSR) (Wold, 1960, Geladi and Kowlaski, 1986) was used to identify the most important combinations of watershed characteristics. PLSR bears some relation to principal components analysis (PCA) in that both techniques are useful for reducing high-dimension multivariate systems that include co-linear variables, but PLSR also exposes the relationships between defined sets of independent and dependent variables, thus PLSR is more useful for prediction (Godoy et al., 2014). In the current context, the independent variables X are the set of watershed characteristics, and the dependent variables Y are the set of flow characteristics (Table 1). The reduced dimensions to which X is transformed are the so-called latent variables. Because these latent variables are linearly related to the watershed characteristics, they can be calculated for all subwatersheds (gauged and ungauged). Further, because PLSR selected the latent variables that best explain the linear relation between watershed and discharge characteristics, they may be used to define hydrological similarity. The PLSR analysis was in this study performed using the R package “pls” (Mevic and Wehrens, 2007).

**Fig. 2:**
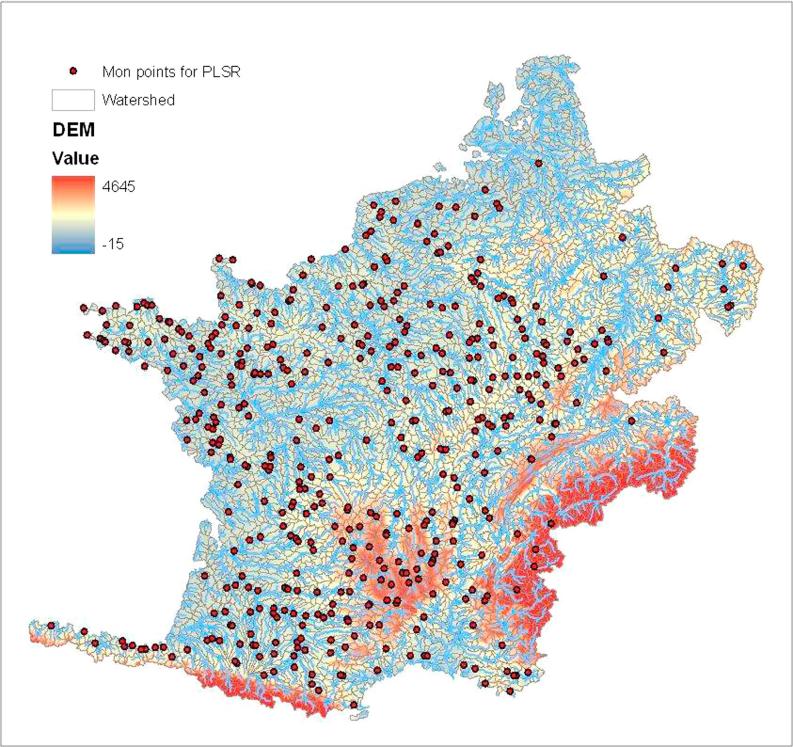
Gauge stations used for PLSR analysis.

From the 698 monitoring points available for this study, 462 spatially distinct watersheds (no upstream gauges) were selected for the PLSR analysis. They include the 246 head subwatersheds selected for calibration plus 216 gauges that are classed as spatially distinct but do not meet the criteria for being in the calibration set. Table A3 further clarifies how the 698 flow gauges were classed and used. Fig. 2 shows the 462 watersheds, they represent a good coverage of the study area.

The variables in the PLSR analysis have different scales, therefore the data for each variable were normalized. This is performed by setting the variance equal to one and the mean equal to zero.

PLSR was performed by adding latent variables into the regression up to the maximum of 19 (equal to the number of watershed characteristics). Each time a latent variable was added, a leave-one-out cross-validation prediction was performed. This involved 462 regressions, each time including 461 data sets with the 462nd used for validation; and calculating the root mean squared error of prediction (RMSEP) over all 462 validation results. The number of latent variables taken forward to the regionalization step was judged based on the rate of improvement in RMSEP.

### 2.7 Regionalization

**Fig. 3:**
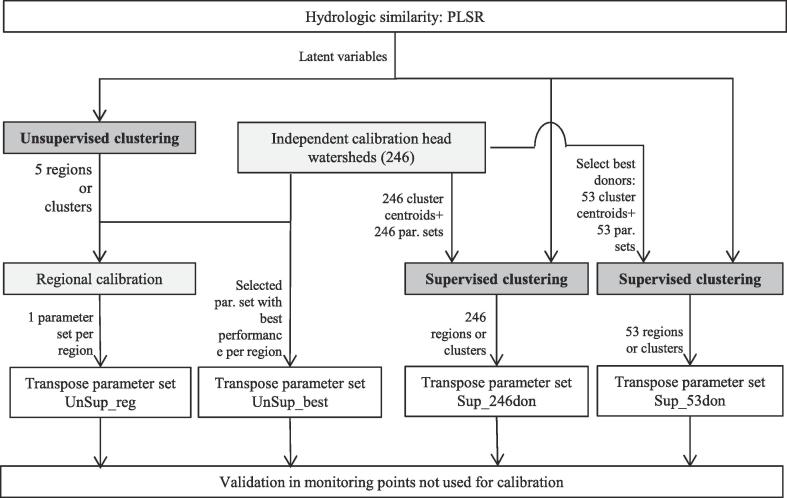
Scheme of regionalization alternatives.

Four alternatives for regionalization based on clustering were investigated (Fig. 3).

#### 2.7.1 Unsupervised clustering

The first two alternatives (*UnSup_reg* and *UnSup_best*) are based on unsupervised clustering of all subwatersheds (both gauged and ungauged) into clusters assumed to be hydrologically homogeneous according to the latent variable space, followed by calibration of gauged subwatersheds (see Section 2.5) to derive a single set of model parameters for each cluster. In method *UnSup_reg* a regional calibration was performed. This involved, for a sampled parameter set, calculating a single objective function that summarizes the performance of all calibration subwatersheds in the cluster. After sampling is complete, this results in a single best parameter set that is then transferred to all ungauged subwatersheds in that cluster. In the method *UnSup_best*, calibration subwatersheds are calibrated individually, after which the calibration subwatershed with the best performance per cluster yields the single parameter set that is transferred to all ungauged subwatersheds in that cluster.

While this clustering method may be considered supervised clustering in the sense that the PLSR and hence the clustering is informed by the hydrological indices, we call it unsupervised clustering because the centroids and number of clusters are not prespecified.

Hierarchical unsupervised cluster analysis was employed using Ward’s minimum variance linkage method (Ward, 1963) together with the Euclidean distance similarity. Ward’s minimum variance criterion minimizes the total within-cluster variance. Initially, all clusters are singletons and, at each step, the two clusters with minimum cluster distance are merged.

Validity indices were used to find the best partitioning to fit the underlying data, i.e. to determine the number of clusters (Kovacs et al., 2005). The corrected Rand index, which measures the level of agreement in cluster membership between clusters, was selected; and also the Meilǎ’s variation of information, which measures the distance between two partitions of the same dataset (Meilǎ, 2007). These were calculated using the “fpc” package in R (Hennings, 2010). The partition selected was that with the lowest corrected Rand index (maximizing the distance between clusters) and the highest Meilǎ’s variation index (minimizing the distances within the cluster).

#### 2.7.2 Supervised clustering

The other two alternatives (Fig. 3, *Sup_246don* and *Sup_53don*) involve supervised clustering around a number of calibrated donor subwatersheds. Each donor subwatershed is predefined as a cluster centroid in the latent variables space, the clustering is performed, and all ungauged subwatersheds that fall into that cluster receive the parameter set of the donor subwatershed. In method *Sup_246don*, all 246 calibrated subwatersheds were used as donors (and cluster centroids), while in method *Sup_53don* only the 53 subwatersheds with the highest calibration performances were used. The k-nearest-neighbour was used as classification rule, using the Euclidean distance function on the latent variables space to be consistent with the unsupervised clustering method. This classification was performed using the “nnr” function of the R package “supclust” (Dettling and Maechler, 2012).

In this work, we refer to this approach as ‘supervised’ because the number of clusters and their centroids are defined *a priori*. This definition differs from the more common view of ‘supervised clustering’ as a modification of standard clustering algorithms to identify class-uniform clusters, i.e., a supervised model selection of unsupervised clustering (e.g. Finley and Joachims, 2005, Michel et al., 2012, Ismaili et al., 2016).

### 2.8 Validation

Validation is done by comparing simulated and observed monthly discharge at the 452 monitoring points with available data that were not used for calibration (Table A3 clarifies how watersheds were classed into calibration and validation watersheds). Goodness of fit parameters were calculated using the R package “hydroGOF” (Zambrano, 2010).

For all four alternatives the calibrated parameters were used for the 246 calibration subwatersheds rather than regionalized values. This is relevant because some of the validation points are downstream of these subwatersheds.

In addition, regionalization performances were compared with those obtained using the parameter values taken from the SWAT library (Neitsch et al., 2009), which are called here the initial parameters. Performances were not compared with those obtainable using calibrated parameters because the validation watersheds generally consist of a large number of subwatersheds and the necessity of a simplified approach to their joint calibration would not necessarily lead to a good benchmark.

To test the regionalization techniques for large aggregations of watersheds and for continental scale applications, the simulated and observed discharges were compared at points close to the outlets of major basins.

## 3 Results

### 3.1 Defining similarity using Partial least squares regression

Table 2 shows the cumulative percentages of the variance of the discharge characteristics explained by adding up to 19 latent variables. It is seen that the watershed characteristics most commonly available (topography, land-use, soil and climate) are not sufficient for fully explaining the hydrological response. For example, in our study, 100% of the variability of the watershed characteristics (see X value) explained only 24.6% of the variability of the coefficient of variation of the annual minima and 26.4% of the ratio baseflow discharge in summer. In general, characteristics describing average conditions (e.g. annual total flow, baseflow and quickflow means) are better explained than characteristics describing extreme events (see e.g. daily discharges with probabilities of exceedance, 50, 70 and 90).

**Table 2:** Cumulative percentage of the variance of the dependent variables explained for each latent variable added in the PLSR model. X represents all the independent variables (watershed characteristics). The variables numbered from 1 to 14 are the dependent variables (discharge characteristics), further explained in Table 1.

Latent variable	1	2	3	4	5	6	7	8	9	10	11	12	13	14	15	16	17	18	19
% variance explained by X	30.5	43.5	50.7	58.2	63.7	67.6	72.2	78.4	82.9	85.5	88.1	92.3	96.3	97.2	98.5	98.9	99.4	99.6	100
1. Annual Qmean	51.4	51.5	66.1	68.5	69.0	70.0	70.4	70.5	70.6	70.7	70.7	70.9	70.9	71.6	71.7	71.7	71.7	71.7	71.9
2. cv of annual Q	17.4	28.2	29.3	29.4	29.5	30.8	30.8	31.0	31.4	31.5	32.0	32.6	32.6	33.1	33.1	33.1	33.2	33.2	33.2
3. cv of daily Q	2.0	22.7	31.3	31.5	31.7	31.8	32.0	32.4	32.4	32.5	33.8	34.4	34.5	36.2	36.2	37.5	37.6	37.6	37.6
4. cv annual min	4.8	11.7	13.3	13.4	18.6	19.5	20.2	22.2	22.2	23.1	23.5	23.6	23.7	24.1	24.1	24.5	24.5	24.5	24.6
5. cv annual max	0.0	25.2	27.3	27.4	27.6	29.1	29.1	29.7	31.2	31.5	31.5	31.6	32.3	32.7	33.0	33.8	34.6	35.0	35.0
6. Qday pbbexc90	25.5	31.3	31.3	38.8	40.9	41.2	41.3	41.3	42.1	42.3	43.0	43.3	43.4	44.0	44.1	44.3	44.4	44.8	44.8
7. Qday pbbexc70	41.4	45.4	46.0	50.4	51.8	52.7	52.8	52.8	53.2	53.2	54.0	54.5	54.5	55.6	55.7	56.2	56.5	57.3	57.3
8. Qday pbbexc50	51.6	54.1	56.9	59.5	60.3	62.2	62.7	62.7	62.8	62.8	63.6	64.0	64.0	65.6	65.6	65.8	66.0	66.6	66.6
9. Annual BFmean	43.0	46.3	49.4	53.8	54.5	56.3	56.4	56.4	56.5	58.1	58.5	58.7	58.7	60.0	60.0	60.0	60.0	60.1	60.4
10. Annual QFmean	39.0	40.0	62.6	63.2	63.3	63.7	64.4	64.5	64.5	64.6	64.9	65.1	65.3	65.4	65.6	65.7	65.7	65.7	65.8
11. BF/Qsummer	0.9	5.7	12.4	14.4	15.3	15.7	18.7	21.3	22.9	25.1	25.2	25.3	25.7	25.7	26.0	26.2	26.3	26.4	26.4
12. BF/Qwinter	0.2	1.6	20.2	24.9	26.0	27.8	29.9	30.8	30.9	33.3	34.4	35.2	35.4	35.9	36.1	36.3	36.4	36.4	36.7
13. BF/PP	30.1	32.5	32.5	35.2	35.8	37.8	37.9	38.1	40.2	43.0	43.3	43.3	43.9	44.4	44.4	44.5	44.6	44.9	45.0
14. QF/PP	32.2	35.1	49.2	49.2	49.2	49.5	50.7	50.8	51.9	51.9	52.5	52.5	52.5	52.6	52.7	52.7	53.1	53.1	53.1

**Fig. 4:**
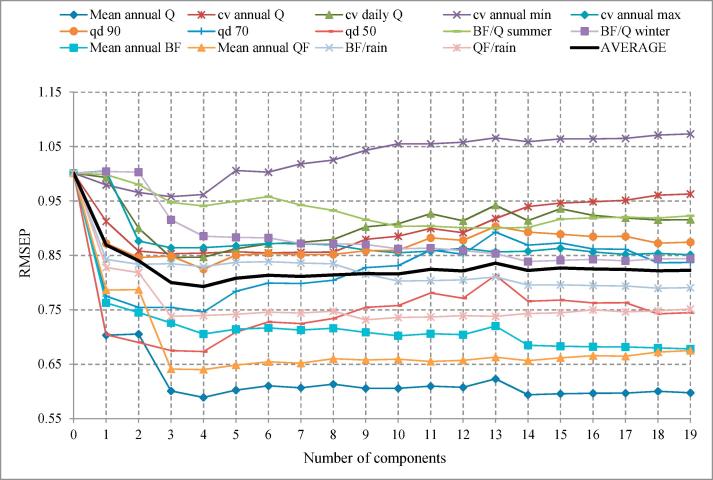
Cross validated root mean squared error of prediction (RMSEP) curves for the independent variables (discharge characteristics).

Fig. 4 shows the corresponding RMSEP values. The starting RMSEP value of 1.0 corresponds to the prediction error using zero latent variables, i.e., the standard deviation of the scaled flow characteristics. From there results, the first four latent variables were considered to explain most of the information contained in the dataset.

Table 3 shows that the first latent variable contains characteristics related to topography, temperatures and percentage of cropland, whereas meteorological characteristics are present in the second and third latent variable and soil properties in the fourth latent variable. The latent variables were calculated for all 4216 subwatersheds (gauged and ungauged) and used as the basis for clustering.

**Table 3:** Loadings of the 4 latent variables (Lat. var.) retained in the PLSR model.

	Lat. var. 1	Lat. var. 2	Lat. var. 3	Lat. var. 4
1. River length			−0.144	0.14
2. Max. Elevation	0.357	−0.264		
3. Min. Elevation	0.307	−0.112	−0.169	−0.462
4. Avg. Elevation	0.372	−0.195	−0.137	−0.184
5. Median slope	0.319	−0.263		0.224
6. Clay	−0.111		0.307	−0.35
7. Sand	0.132		−0.215	0.263
8. Shrub		−0.204	0.279	0.237
9. Bare soil				0.288
10. Forest	0.274	−0.306		0.106
11. Water	0.131		−0.15	
12. Urban		0.147	−0.199	0.515
13. Cropland	−0.338	0.122		
14. Grassland		0.297	0.283	−0.381
15. Annual precipitation	0.257		0.548	0.153
16. Avg. Max. Temperature	−0.322	−0.301	0.184	0.148
17. Avg. Min. Temperature	−0.345	−0.1	0.314	0.292
18 Annual potential evapotranspiration	−0.179	−0.525		
19. Avg. Rainy days	0.127	0.497	0.391	

### 3.2 Unsupervised clustering – UnSup_reg and UnSup_best

**Fig. 5:**
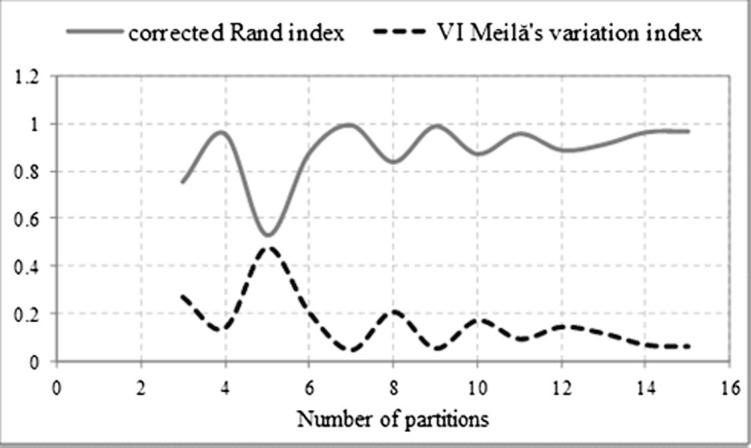
Corrected Rand and Mielă’s variation indices per number of partitions.

**Fig. 6:**
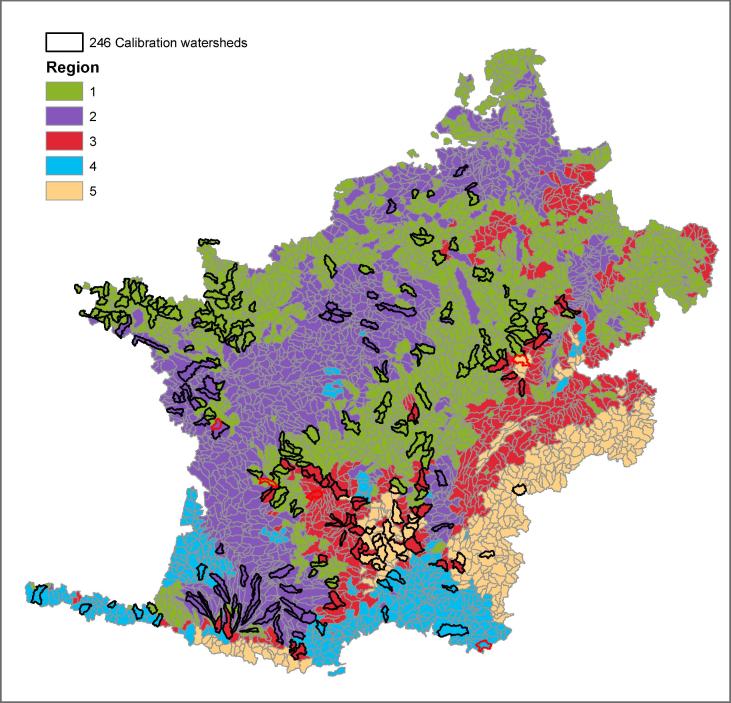
Hydrological regions obtained after cluster analysis and the 246 calibration subwatersheds. In red calibration subwatersheds with the best performance after calibration used in *UnSup_bes* method. (For interpretation of the references to colour in this figure legend, the reader is referred to the web version of this article.)

Fig. 5 shows the corrected Rand and Meilă’s indices for different numbers of clusters, from which five partitions were selected. At this point the corrected Rand index is minimised indicating dissimilarity between clusters and at the same time the Meilă’s index is maximised indicating a good linkage between cluster members. The resulting hydrological clusters are presented in Fig. 6. Most of these regions are spatially interconnected; and, moreover, comparison with Fig. 1 shows that the resulting hydrological regions are consistent with topography and land-use maps.

The spatially distinct head subwatersheds used for calibration are also shown in Fig. 6. The numbers of calibration watersheds are 118, 55, 41, 13 and 19 for regions 1, 2, 3, 4, and 5, respectively. The calibrated subwatersheds with the best performance per hydrological region (*UnSup_best)* are indicated in red.

Table 4 compares the performances of *UnSup_reg and UnSup_best* in the validation watersheds. Both regionalization methods perform better than when using the initial (INI) parameters. For example, bR^2^ equals 0.54 when the initial parameters are used and 0.65 and 0.61 using *UnSup_reg* and *UnSup_best*. *UnSup_reg* performs better than *UnSup_best* for the Normalized Root Mean Squared Error (NRMSE) but, on average, it underestimates the discharge.

**Table 4:** Model performance for the 4 regionalization methods (*UnSup_reg*, *UnSup_best*, *Sup_246don*, *Sup_53don*) and initial (INI) parameter values at 452 monitoring points used for validation, of 167 watersheds with more than 50% presence of karstic areas and of 285 watersheds with no presence, or less than 50%, of karstic area. Performance indicators compare monthly simulated and measured discharges for the period 1995–2004.

		452 monitoring points used for validation					167 of 452 monitoring points used for validation with more than 50% of karst area					285 of 452 monitoring points used for validation with less than 50% of karst area				
		INI	*UnSup_reg*	*UnSup_best*	*Sup_246don*	*Sup_53don*	INI	*UnSup_reg*	*UnSup_best*	*Sup_246don*	*Sup_53don*	INI	*UnSup_reg*	*UnSup_best*	*Sup_246don*	*Sup_53don*
bR2	AVG	0.54	0.65	0.61	0.64	0.63	0.50	0.64	0.63	0.64	0.63	0.55	0.65	0.59	0.65	0.62
	MAX	0.78	0.90	0.92	0.91	0.90	0.78	0.90	0.92	0.90	0.90	0.78	0.90	0.90	0.91	0.90
	MIN	0.07	0.02	0.05	0.14	0.05	0.11	0.07	0.05	0.24	0.10	0.07	0.02	0.05	0.14	0.05
	ST.DEV	0.14	0.15	0.15	0.13	0.15	0.14	0.14	0.15	0.13	0.13	0.13	0.15	0.16	0.14	0.16

NRME%	AVG	87.7	65.3	75.8	61.7	65.2	107.6	70.2	74.9	65.6	68.3	76.1	62.5	76.3	59.4	63.4
	MAX	766.9	303.3	338.0	140.3	240.5	766.9	303.3	338.0	140.3	240.5	215.3	202.2	214.5	139.7	216.6
	MIN	41.3	24.8	24.8	24.8	24.8	41.3	24.8	24.8	24.8	24.8	43.4	29.8	29.0	27.6	27.6
	ST.DEV	59.4	28.7	35.1	20.5	25.3	86.1	31.8	39.1	21.3	25.3	30.0	26.4	32.6	19.7	25.1

PBIAS%	AVG	26.7	12.4	−0.6	7.1	8.4	44.7	20.9	12.8	16.0	19.8	16.1	7.5	−8.5	1.8	1.6
	abs(MAX)	398.3	170.7	130.8	107.8	140.3	398.3	170.7	130.8	107.8	137.5	144.8	118.9	98.5	102.0	140.3
	abs(MIN)	0.1	0.0	0.0	0.1	0.1	0.6	0.1	0.1	0.1	0.1	0.1	0.0	0.0	0.1	0.1
	ST.DEV	49.0	30.6	31.7	29.6	33.7	62.4	30.3	31.0	29.3	31.0	35.0	29.8	29.4	28.5	33.4

bR^2^: weighted R^2^ explained in Section 2.5, NRMSE%: Normalized Root Mean Squared Error, PBIAS%: Percent Bias; AVG = average over all included catchments; MAX = maximum over all included catchments; MIN = minimum over all include catchments; ST.DEV = −standard deviation over all included catchments.

The average of the 246 calibration performances, the averages of the pooled calibration per cluster (for *UnSup_reg* alternative), and the best calibration performance per cluster (for *UnSup_best* alternative) are presented in Table A2 in the appendices.

### 3.3 Supervised clustering – Sup_246don and Sup_53don

Using Sup_*246don*, where all 246 calibrated subwatersheds were used as cluster centroids, the size of clusters varied from 1 to 99, i.e. there were clusters consisting of solely the calibrated subwatershed, therefore not resulting a suitable donor to any ungauged subwatershed.

For the *Sup_53don* method, from the 246 possible donor subwatersheds, only those with a NRMSE lower than 50% were used. This resulted in 53 donor subwatersheds, with cluster sizes from 1 to 335.

Table 4 shows that, at the validation points, *Sup_246don and Sup_53don* perform better than the initial (INI) parameters. For example, bR^2^ is 0.64 for *Sup_246don* and 0.63 for *Sup_53don* against 0.54 using the initial parameters. Analysing all the performance indicators, *Sup_246don* performs better than *Sup_53don*.

### 3.4 Comparing performance of the regionalization methods

**Fig. 7:**
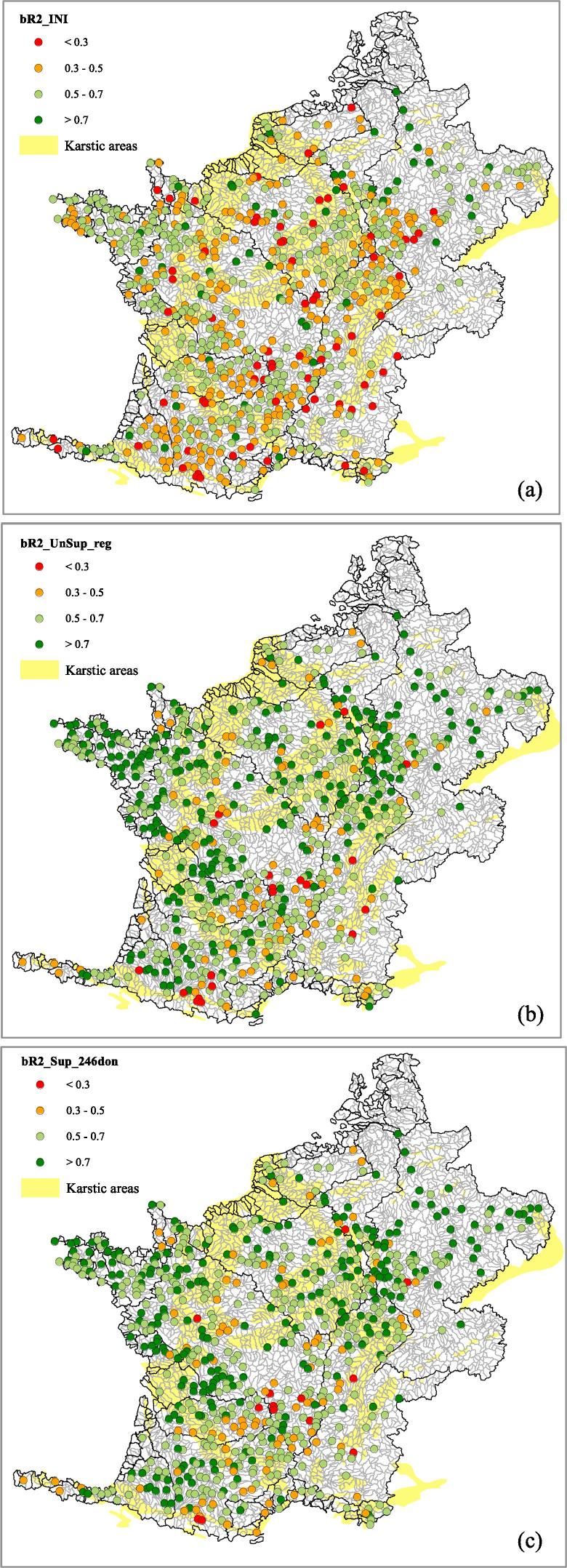
Average monthly bR^2^ efficiencies for regionalization alternatives in all 698 monitoring points for calibration period 1995–2004 for (a) initial (INI) parameters, (b) *UnSup_reg* and (c) *Sup_246don*.

The regionalization methods with the best performance are *Sup_246don* and *UnSup_reg*, which both transferred information from all 246 subwatersheds, in contrast with *Sup_53don* and *UnSup_best* methods, which used only a subset of the 246 subwatersheds based on calibration performances. *Sup_246don* has lowest standard deviation of bR^2^ over all validation subwatersheds (Table 4) as well as the lowest minimum value of bR^2^; also, as well as having the lowest average NRMSE%, it has by far the lowest standard deviation of NRMSE%. There is some evidence therefore that Sup_246don gives less variable performance than *UnSup_reg.* The bR^2^ performance of *Sup_53don* (53 donor subwatersheds), although lower than that of *UnSup_reg*, only decreases by *circa* 2% (from 0.65 to 0.63 for the validation watersheds) while using only 22% of the available donor watersheds. This result suggests that the supervised clustering method is more efficient in using information, at least when the model performs well in the selected donor subwatersheds. Fig. 7 shows the spatial variability of bR^2^ across all 698 (calibration and validation) monitoring points for *Sup_246don* and *UnSup_reg*, and it can be seen that simulations improved throughout the study area.

Predictions were affected by the presence of karst formations. According to the Geological Map of Europe they are present in approximately 25% of the modelled domain and are distributed throughout the area (indicated in yellow in Fig. 7). Subwatersheds containing more than 50% of karst areas were not considered for regional calibration or as donor subwatersheds, but they were included in the validation. We separated the performance of the validation watersheds that are more than 50% karstic (Table 4). It is seen that results using initial parameters are worse for the karstic watersheds. The regionalization improved the simulations, more so for the non-karstic watersheds. Results for percent bias (PBIAS%) suggest that on average in karstic watersheds simulated discharges are underestimated (Table 4).

Performances at basin scale were found to be less variable than at smaller watersheds, and a little higher on average than over the full validation data set, which may be presumed to be due to integration of random errors in the data and parameter estimates. Table 5 lists the monitoring points located near the outlet of the major continental-scale basins and their performances using *UnSup_reg* and *Sup_246don*. Results show that for the Rhine, the Garonne, the Dordogne, the Vilaine and the Rhone, *UnSup_reg* performs better (although for the Rhone the initial parameter estimates are the best) while *Sup_246don* performs better for the Loire and the Adour and both alternatives perform similarly for the Loire and the Adour. The continental scale catchments – with exceptions of the highly karstic Dordogne and Rhone- have better performances than the average found in the subwatersheds.

**Table 5:** Model performance for the best two regionalization techniques (*UnSup_reg* and *Sup_246don*) at monitoring points closer to the outlet of the major basins modelled comparing monthly simulated and measured discharges for the calibration period 1995–2004.

Basin	Monitoring point	Basin area (km2)	Fraction Karst area	Fraction Calibrated	INI			*UnSup_reg*			*Sup_246don*		
					bR2	NRMSE%	PBIAS%	bR2	NRMSE%	PBIAS%	bR2	NRMSE%	PBIAS%
Rhine	River Rhine at Rees	160,221	0.03	0.03	0.72	61	5.5	0.82	44.9	2.4	0.78	47.1	−6.6
Loire	River Loire at Nantes	116,981	0.28	0.14	0.72	55.3	29.3	0.79	43.7	16.3	0.82	39.5	7.0
Rhone	River Rhone at Beaucaire	96,619	0.32	0.07	0.66	56.1	−15.2	0.57	62.2	−13.9	0.64	65.8	−23.7
Seine	River Seine at Rouen	75,990	0.53	0.08	0.75	53.4	14.7	0.79	50.5	2.2	0.79	40.0	−7.2
Garonne	River Garonne at Tonneins	55,703	0.17	0.17	0.74	43.4	−3.1	0.74	40.5	−9.6	0.72	42.4	−14.7
Meuse	River Meuse at Sint Pieter noord	32,047	0.14	0.09	0.74	54.3	21.5	0.90	35.2	13.9	0.88	35.1	11.9
Dordogne	La Dordogne à Pessac-sur-Dordogne	23,902	0.33	0.10	0.51	65.9	−26.6	0.58	62.9	−33	0.50	75.2	−39.1
Adour	River Adour at Sint Vincent de Paul	16,861	0.27	0.19	0.64	67.3	24.8	0.74	52.5	20.4	0.74	39.3	−10.4
Vilaine	La Vilaine à Rieux	10,490	0.00	0.13	0.58	52.7	2.9	0.71	37.5	−9.4	0.62	46.8	−4.6

bR^2^, NRMSE% and PBIAS% are explained in Table 4.

**Fig. 8:**
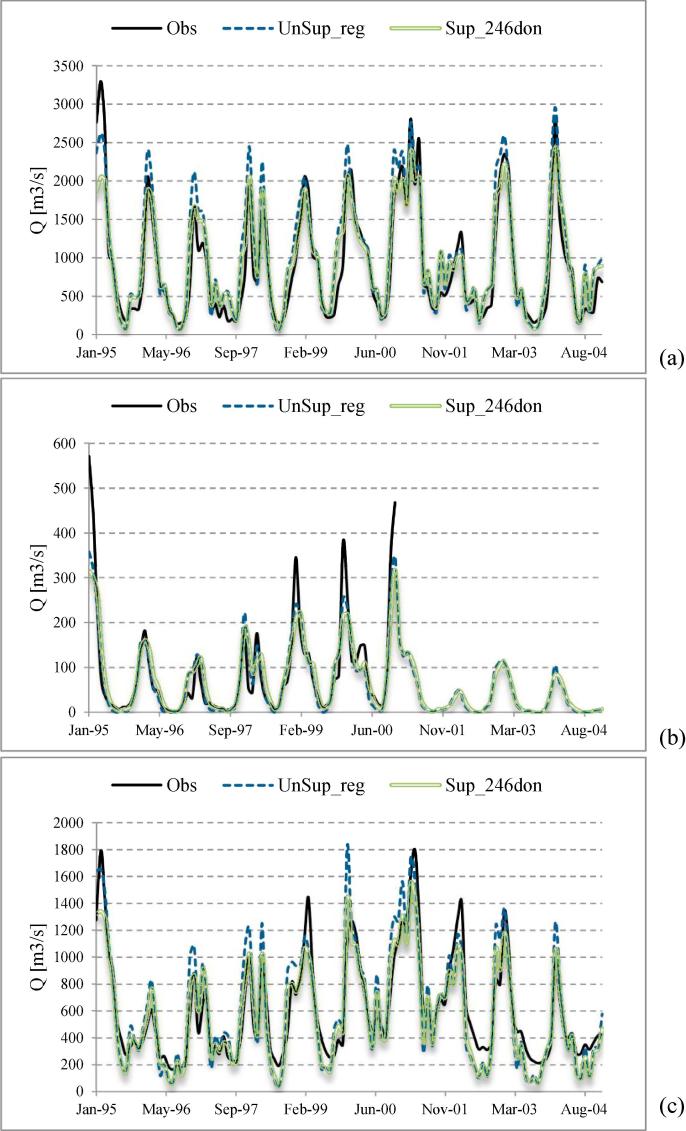
Observed and simulated monthly discharge for calibration period 1995–2004 for (a) Loire basin, (b) Vilaine basin, (c) Seine basin.

Fig. 8 shows the simulated versus observed monthly hydrographs for the Loire, the Vilaine and the Seine river basins. For the Loire river basin (Fig. 8a) the case *Sup_246don* performs better, and for the Vilaine river basin (Fig. 8b) the simulated discharge for the *UnSup_reg* alternative has a better agreement with the observed one. When we compare these results for the Seine river basin (Fig. 8c), both alternatives perform similarly. However, for that basin, the *Sup_246don* alternative simulates slightly better the shape of the discharge signal, whereas alternative *UnSup_reg* simulates better the peaks.

## 4 Discussion

This paper investigates alternative regionalization techniques for continental scale hydrological modelling. Although the SWAT model was used, the regionalization methods used are independent of the chosen hydrological model.

The regionalization techniques are based on the similarity approach, which transfers the calibrated parameter sets of gauged watersheds to similar ungauged watersheds. In our work, similarity is defined by a combination of observable watershed physical characteristics that are linearly related to a selected set of hydrologic signature indices as defined by PLSR. This removes much of the subjectivity in selecting similarity metrics.

However, the similarity metrics will depend on the selected hydrological signatures. In this study, as the final objective of the hydrological model is to simulate both water quantity and water quality, the latter depending on multiple aspects of the flow regime, the hydrological indices used cover a wide range of hydrological conditions. The selection of the hydrological characteristics, however, is not fixed and used must be in agreement with the project objectives. The identification of similarity metrics is also restricted by the linearity assumption in the PLSR, although this may be addressed by exploring suitable non-linear transforms of the variables.

It was shown that the watershed characteristics most commonly available (topography, land-use, soil and climate) are not sufficient for fully explaining the hydrological response, especially flow variability and low flows. Similar findings were obtained by Merz and Blöschl, 2004, Merz and Blöschl, 2005, Merz et al., 2006. These results are also in agreement with the results found in Oudin et al. (2010) and suggest that additional watershed characteristics may need to be incorporated. This is likely to include hydrogeologic characteristics and/or watershed flow regulation characteristics – data that are not available over the broad scale of this study. Karst was isolated as a particular potential issue and results show evidence that performance was overall worse in more karstic watersheds (Table 4). When integrating to large catchment scale (Table 5), performance was poor in two catchments with high presence of karst, although relatively good in the Seine, which has the highest presence of karst.

The relative success of the *UnSup_reg* alternative supports the view that suitable model parameters can be obtained if a number of gauged watersheds in a region (in this case in a region defined by similarity) are used simultaneously in a regional calibration. Although the value of regional calibration, with its goal of modelling regional trends rather than local hydrology, is still controversial (Parajka et al., 2007), the present study shows that regional calibration is at least as successful as alternative regionalization approaches and is potentially useful in continental-scale modelling, where calibration options are severely constrained by data, time and computational resources.

In the supervised clustering approach, the number of clusters and their centroids are defined *a priori*, but results showed that after the clustering process there were available donor subwatersheds that were discarded by the data, i.e., resulted in clusters with only one member; the calibrated subwatershed. This indicates that, in this approach, ungauged subwatersheds cluster towards the most suitable donors and discard those that are not. Further, the discarded subwatersheds are not forced to join any cluster and remain with their calibrated parameters. This approach results in more variability in the regionalized calibrated parameters.

Using the regionalization techniques investigated here, we avoid the potential propagation of errors downstream that can be present in more standard calibration procedures. In most nested calibration studies, a basin is calibrated in steps starting with the headwater watersheds, then moving downstream to monitoring points carrying along calibration errors and uncertainties. This may lead to overcalibration to compensate for errors in the downstream watersheds, e.g. due to hydraulic influences ignored by the model. In addition, the use of the similarity approach identifies watersheds with different hydrological behaviours and assigns different parameter sets accordingly, a fact that is often overlooked in typical calibration procedures where the entire upstream drainage area is calibrated to match the discharge at the outlet. Whether the accuracy of flows in individual types of watershed is important or not depends on the application; but even in a continental-scale application, the potential dependence of water quality on local features supports the need for parameter estimation at the smallest possible scale supported by the data.

Results of the techniques defining hydrological regions *a priori* and *a posteriori* are similar (e.g. results for *UnSup_reg* versus *Sup_246don* in Table 4, Table 5). Reasons behind the relative success of one regionalization method for a particular basin are not clear. Analysis of results with both methods do not indicate any consistency or aggregation of errors with scale. We believe that the selection of the regionalization technique is case specific and depends largely on the amount and quality of available data.

As well as the uncertainty that inevitably arises when selecting and calibrating a hydrological model (Beven, 2006), significant uncertainty is likely to arise from the regionalization process. Uncertainty exists in the hydrologic signature and physiographic characteristic estimates; there are assumptions and subjectivity involved in selecting signatures and in the PLSR and the clustering; and the impact of unavailable but theoretically relevant physiographic characteristics is unknown. The relative success of the alternative regionalisation approaches is likely to be related to the relative importance of these sources of uncertainty, and their relative importance compared to model equifinality and rainfall and flow observation uncertainty. Recommended extension of this work is to explicitly quantify the uncertainties and conduct a sensitivity analysis that provides greater insight into which of them is affecting choice of regionalization procedure.

## 5 Conclusions

Four regionalization techniques were investigated in the context of pan-European hydrological modelling. Each technique aimed to regionalize the information in relatively small and non-overlapping gauged subwatersheds to give flow predictions at multiple scales up to the largest continental-scale rivers. Each technique used the similarity approach to regionalization. The watershed characteristics defining similarity were selected based on their degree of relevance to selected hydrological signature indices, identified using Partial Least Squares Regression. Similar watersheds were defined through advanced clustering. The first two techniques define the hydrological regions a priori based only on the watershed characteristics (termed “unsupervised clustering”); while the second two techniques define the regions a posteriori, after model calibration, with the clusters centred on gauged sub-watersheds (termed “supervised clustering”). Although the SWAT model was considered appropriate due to the prior effort invested in its calibration, and the breadth and depth of experience of SWAT that exists in Europe, all four regionalization methods can be applied to any hydrological model.

Results showed that the supervised clustering performed better overall. For either supervised or unsupervised clustering, using all available gauged subwatersheds performed better than using only a sub-sample, as may be expected. However, the overall performance of the supervised clustering was good even when using the 53 gauged subwatersheds with the best performance in calibration. This suggests that when available information is scarce, supervised clustering can give acceptable results.

Using the proposed regionalization techniques the available data for calibration were efficiently used. Even when including all gauged subwatersheds, only 10% of the modelled area was calibrated, and the performance at the larger scale validation points significantly improved across all the performance measures relative to using the initial SWAT model parameters. Although model performances were considered good overall, it was shown that watershed characteristics most commonly available (topography, land-use, soil and climate) are not always sufficient, and additional information should ideally be included, with priorities being karst and other hydrogeological properties, and flow regulation data.

## Figures and Tables

**Table A1:** Model parameters.

File	Parameter	Description	Type
SNOW	SFTMP	Snowfall temperature [⁰C]	C
	SMTMP	Snow melt base temperature [⁰C]	C
	SMFMX	Melt factor for snow on June 21 [mm H_2_O/⁰C-day]	C
	SMFMN	Melt factor for snow on Decembre 21 [mm H2O/⁰C-day]	C
	TIMP	Snow pack temperature lag factor	C
	SNOCOVMX	Minimum snow water content that corresponds to 100% snow [mm H2O]	D
	SNO50COV	Fraction of snow volume representes by SNOCOVMX that corresponds to 50% snow cover	D

SBN	SURLAG	Surface runoff lag coefficient	D

SUB	SUB_KM	Area of subbasin [km2]	I
	SUB_ELEV	Elevation of subbasin [m]	I
	ELEVB	Elevation at the center of the elevation band [m]	pC
	ELEV_FR	Fraction of subbasin area within the elevation band	pC
	PLAPS	Precipitation lapse rate [mm H2O/km]	C
	TLAPS	Temperature lapse rate [⁰C/km]	C
	CH_L(1)	Longest “tributary” channel length in subbasin [km]	I
	CH_S(1)	Average slope of tributary channels [m/m]	I
	CH_W(1)	Average width of tributary channels [m]	I
	CH_K(1)	Effective hydraulic conductivity in tributary channel alluvium [mm/hr]	D
	CH_N(1)	Manning's “n” value for the tributary channels	D

CROP	ICNUM	Land cover/plant code	I
	T_OPT	Optimal temperature for plant growth [⁰C]	pC
	T_BASE	Minimum (base) temperature for plant growth [⁰C]	pC

HRU	SLSUBBSN	Average slope length [m]	I
	HRU_SLP	Average slope steepness [m/m]	I
	CANMX	Maximum canopy storage [mm H2O]	D
	OV_N	Manning's value for overland flow	D
	LAT_TIME	Lateral flow travel time [days]	I
	EPCO	Plant uptake compensation factor	C
	ESCO	Soil evaporation compensation factor	C

MGT	PLANT_ID	Land cover identification number	I
	CN2	Initial SCS runoff curve number for moisture condition II	C
	MGT_OP	Management operation number	pC
	HUSC	Fraction of total base zero heat units at which operation takes place	pC
	CURYR_MAT	Current age of tress [years]	pC
	HEAT UNITS	Total heat units for cover/plant to reach maturity	pC
	WSTRS_ID	Water stress identifier (for trigger autoirrigation)	pC
	AFERT_ID	Fertilizer identification number from fertilizer database	pC
	AUTO_NSTRS	Nitrogen stress factor of cover/plant that triggers fertilization	pC
	FRT_KG	Amount of fertilizer applied to HRU [kg/ha]	pC

SOL	HYDGRP	Soil hydrologic group	I
	SOL_Z	Depth from soil surface to bottom of layer [mm]	I
	SOL_BD	Moist bulk density [Mg/m3]	I
	SOL_AWC	Available water capacity of the soil layer [mm H2O/mm soil]	C
	SOL_K	Saturated hydraulic conductivity [mm/hr]	C
	SOL_CBN	Organic carbon content [% soil weight]	I
	SOL_CLAY	Clay content [% soil weight]	I
	SOL_SILT	Silt content [% soil weight]	I
	SOL_SAND	Sand content [% soil weight]	I
	SOL_ROCK	Rock fragment content [% soil content]	I
	SOL_ALB	Moist soil albedo	I
	USLE_K	USLE equation soil erodibility	I

GW	GW_DELAY	Groundwater delay time [days]	C
	ALPHA_BF	Baseflow alpha factor [days]	C
	GWQMN	Threshold depth of water in the shallow aquifer required for return flow to occur [mm H2O]	C
	GW_REVAP	Groundwater “revap” coefficient	C
	REVAPMN	Threshold depth of water in the shallow aquifer for “revap” or percolation to the deep aquifer to occur [mm H2O]	C
	RCHRG_DP	Deep aquifer percolation fraction	C

RTE	CH_W(2)	Average width of main channel at top bank [m]	I
	CH_D	Depth of main channel from top of bank to bottom	I
	CH_S(2)	Average slope of main channel along the channel length [m/m]	I
	CH_L(2)	Length of main channel [km]	I
	CH_N(2)	Manning's “n” value for the main channel	D
	CH_K(2)	Effective hydraulic conductivity in main channel alluvium [mm/hr]	D

Type “C” parameters correspond to those calibrated; “D” to default model parameters; “I” to those estimated from distributed input data and “pC” to those adjusted prior calibration related crop management.

**Table A2:** Calibration performance indicators: averages for individual calibration of 246 calibration subwatersheds, regional calibration per cluster for the *UnSup_reg* method and calibration subwatershed with the best performance per cluster for the *UnSup_best* method. Performance indicators compare monthly simulated and measured discharges for the calibration period 1995–2004.

Regionalization alternative		All	UnSup_reg					UnSup_best				
Cluster		–	1	2	3	4	5	1	2	3	4	5
Nr calibration subwatersheds		All 246	118	55	41	13	19	best of 118	best of 55	best of 41	best of 13	best of 19
bR2	INI	0.47	0.51	0.44	0.4	0.44	0.41	0.55	0.57	0.54	0.5	0.69
	CAL	0.63	0.6	0.51	0.47	0.46	0.43	0.87	0.85	0.8	0.68	0.81
NRMSE%	INI	97.46	82.3	127.8	101.4	98	94.9	92	65.7	74.2	97	47.9
	CAL	64.8	75.8	119.5	96.2	122.4	92.3	33.7	37.5	48.2	57.5	34.2
PBIAS%	INI	31.8	28.8	64.4	5.9	25.2	16.3	56.5	18.4	24.5	99.6	−0.9
	CAL	11.2	24.2	42.8	−3.1	41.2	18.2	5	0.2	3.3	26.6	−3.8

bR^2^, NRMSE% and PBIAS% are explained in Table 4

**Table A3:** Use of monitoring points.

Gauged areas consisting of one or more subwatersheds **698**	Spatially distinct gauged areas consisting of one or more subwatersheds (no upstream gauges) **462**	Spatially distinct gauged areas that are single subwatersheds **246**	PLSR	Calibration
		Other spatially distinct gauged areas **216**		Validation
	Not spatially distinct areas consisting of one or more subwatersheds (contain upstream gauged units) **236**			

## Appendix

### A.1. Calibration parameters and calibration results

Calibrated parameters related to flow simulation are presented in Table A1. For the calibration parameters “C”, calibration ranges were set considering authors experience and literature recommendations but for parameters related to properties that were interpolated from input data, as CN2, SOL_AWC and SOL_K, they were varied as a percentage of their original values, to maintain heterogeneity.

Performance metrics for the average of the individual calibration of the calibration subwatersheds, for the averages of the pooled calibration per cluster (for *UnSup_reg* alternative) and for the calibration subwatershed with the best performance per cluster (for *UnSup_best* alternative) are presented in the Table A2.

Results of the average performance of the individual calibration of the calibration subwatersheds indicates that bR^2^ increased from 0.46 with the initial conditions to 0.63 after calibration. When calibrating calibration subwatersheds pooled per region (*UnSup_reg*), performance results, as expected, are lower, increasing bR^2^ indicator from 0.51 with initial conditions to 0.60 for cluster 1, from 0.44 to 0.51 for cluster 2, from 0.40 to 0.47 for cluster 3, from 0.44 to 0.46 for cluster 4 and from 0.41 to 0.43 for cluster 5. Performance indicators of the selected calibration subwatershed per cluster for the *UnSup_best* alternative are above a bR^2^ of 0.80 for all regions except for region 4 that is 0.68 (Table A2).

### A.2. Use of gauged data

Table A3 synthetizes the use of monitoring points data.
